# A Meta-Analysis of the Characterisations of Plastic Ingested by Fish Globally

**DOI:** 10.3390/toxics10040186

**Published:** 2022-04-11

**Authors:** Kok Ping Lim, Phaik Eem Lim, Sumiani Yusoff, Chengjun Sun, Jinfeng Ding, Kar Hoe Loh

**Affiliations:** 1 Institute for Advanced Studies, Universiti Malaya, Kuala Lumpur 50603, Malaysia; kokping@um.edu.my; 2 Institute of Ocean and Earth Science, Universiti Malaya, Kuala Lumpur 50603, Malaysia; sumiani@um.edu.my (S.Y.); khloh@um.edu.my (K.H.L.); 3 Key Laboratory of Marine Eco-Environmental Science and Technology, Marine Bioresource and Environment Research Centre, First Institute of Oceanography, Ministry of Natural Resources, Qingdao 266061, China; csun@fio.org.cn (C.S.); dingjf1004@gmail.com (J.D.)

**Keywords:** microplastic, shape, colour, polymer type

## Abstract

Plastic contamination in the environment is common but the characterisation of plastic ingested by fish in different environments is lacking. Hence, a meta-analysis was conducted to identify the prevalence of plastic ingested by fish globally. Based on a qualitative analysis of plastic size, it was determined that small microplastics (<1 mm) are predominantly ingested by fish globally. Furthermore, our meta-analysis revealed that plastic fibres (70.6%) and fragments (19.3%) were the most prevalent plastic components ingested by fish, while blue (24.2%) and black (18.0%) coloured plastic were the most abundant. Polyethylene (15.7%) and polyester (11.6%) were the most abundant polymers. Mixed-effect models were employed to identify the effects of the moderators (sampling environment, plastic size, digestive organs examined, and sampling continents) on the prevalence of plastic shape, colour, and polymer type. Among the moderators, only the sampling environment and continent contributed to a significant difference between subgroups in plastic shape and polymer type.

## 1. Introduction

Global plastic production has increased drastically from around 1.5 million tonnes in 1950 to 368 million tonnes in 2019, due to the high demands of consumers [1,2]. As a consequence of the large production volume of plastics and defective waste management system, it is very common for plastics to accumulate in the environment, such as in seawaters [3,4], deep sea sediments [5], artic sea ice [6], lakes [7], soils [8], and even in the atmosphere [9]. Slow degradation of the plastics has led to their accumulation in the environment. Nonetheless, radiation, heat and friction may cause fragmentation of the plastics [4] and turn them into secondary microplastics, which are plastic particles less than 5 mm in size [10]. Additionally, primary microplastics are produced purposefully to be used in various products [11] or industries [12].

It is estimated that between 1.15 and 2.41 million tonnes of mismanaged plastic waste are discharged into the oceans through rivers annually [13]. In 2014, it was estimated that at least 5.25 trillion plastic particles, weighing 268,940 tonnes, were floating in the world’s oceans [14]. Hence, there is an increased risk of marine organisms ingesting plastic particles due to their high concentration in oceans. Organisms might ingest the particles by primary ingestion because they recognise the items as potential prey, or secondary ingestion via contaminated prey [15]. Many publications have shown that plastic particles are ingested by a wide variety of animal taxa in various environments, including seabirds [16], waterbirds [17], crustaceans [18,19], sharks [20] and other fish [21] and cetaceans [22,23]. Furthermore, there is trophic transfer in the ecosystem from lower to higher trophic level based on both experimental [24,25] and field studies [26,27,28,29].

Direct fatality due to the blockage of the digestive tract by larger size plastic debris has been found in many marine organisms, such as turtles [30], sea birds [31], and manatees [32]. The death of a whale shark was suspected to be caused by plastic ingestion with subsequent inflammation of the stomach mucosa triggering wounds and infections [33]. Several severe impacts due to the ingestion of plastic particles by fish in laboratory conditions have also been documented [34,35]. The plastic particles are able to promote inflammation and accumulation of lipids in zebrafish liver [36]. The growth and body condition of reef fish decreased significantly when food pieces were substituted by microplastic particles, and these effects escalated at higher microplastic concentrations [37]. Intestinal lesions in fish were observed in an experimental study and the severity increased with the concentration of microplastics [38]. Nevertheless, the exposure settings for the laboratory experiments cannot fully represent the natural environments in which the plastic types, sizes, and concentrations may fluctuate temporally and spatially.

Plastic ingestion by fish has been fairly well reviewed. The earliest review reported the incidence of plastic ingestion in 22 fish species [39]. Subsequent and more recent reviews have recorded the number of fish as follows: 90 species [40], 34 [41], 95 [42], 200 [43], 323 [44], 165 [45]; and 386 [46]. There were also various reviews on plastic ingestion by fish, but these included other marine biotas [47,48,49]. A systematic review of the occurrence of microplastics based on their characterisations was conducted but limited to freshwater fish species [50]. In view of the gaps in the knowledge on plastic characterisations in different environments, a meta-analysis, which included samples from all environments, was conducted to investigate the possible factors affecting plastic ingestion by fish, and to identify the abundance of plastic ingested on a global scale based on its characterisations.

## 2. Materials and Methods

### 2.1. Literature Review

In this review paper, a literature review was conducted using web-based search engines: Google Scholar and electronic databases, such as PubMed, Web of Science, Science Direct and Wiley Online Library from 1970 to December 2021 with the following keywords: “microplastic” OR “plastic” OR “plastic ingestion” OR “marine debris” AND “fish”.

### 2.2. Quality Assessment and Data Extraction

**Figure 1:**
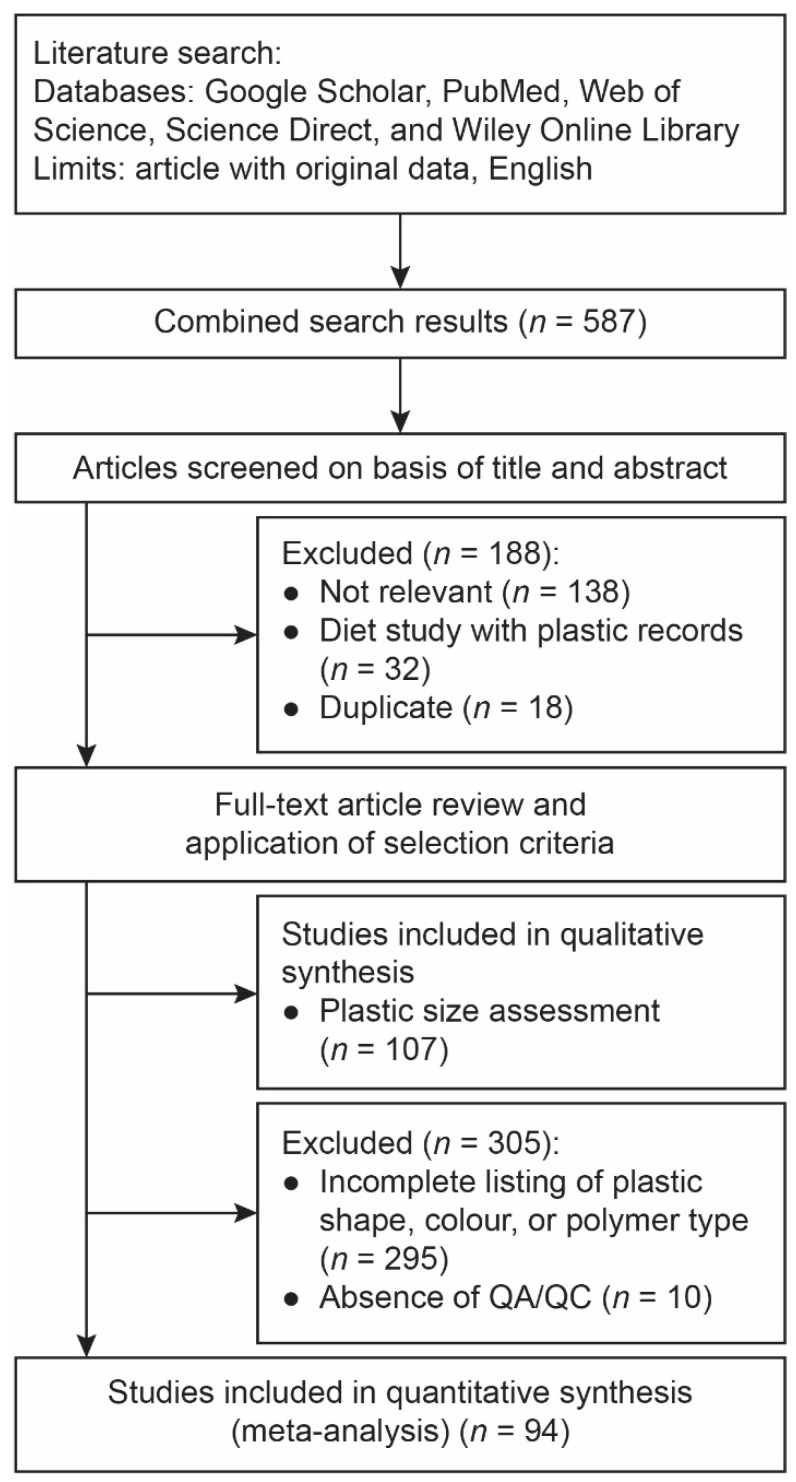
Flow diagram of study selection.

The publications were reviewed based on the following criteria (Figure 1). Firstly, the titles and abstracts of the articles were screened to search for related studies. Studies on fish exposure to plastics in a laboratory setting were excluded. In the second step, the materials and methods section of each article was examined to ensure that the numbers of plastic shape, colour, and polymer type were reported. If the data were not reported in numbers, they were extracted from published diagrams using WebPlotDigitizer Version 4.5 (Ankit Rohatgi, Pacifica, CA, USA). Studies that assigned plastic size class and predominant size class were included for qualitative analysis. Due to the importance of contamination control in plastic research during the extraction process, the studies were checked for quality assessment/quality control (QA/QC). Studies that did not include any QA/QC were excluded from meta-analysis of plastic characterisation.

Detailed data-location, part of digestive organs examined, plastic extraction method, percentage of plastic ingested, plastic size, shape, and colour, and the polymer type were recorded. The environments where the samples were collected were retrieved from the publications based on the GPS coordinates given or sampling procedures stated in the method in each publication. The source of the samples was classified into marine, estuary, freshwater, aquaculture, and market. Samples obtained from markets were grouped into marine, estuary, or freshwater if the study specified the source of the samples [51]. Studies that purchased samples directly from the market without the source information of the samples were classified into the “market” category [52]. The plastic extraction methods were categorized into three groups, as proposed by a previous review [44]. Method 1 is a visual analysis of the GIT content with the naked eye; Method 2 is a visual analysis of the GIT content using a microscope; and Method 3 is the chemical digestion of the GIT content, followed by filtration and microscope analysis. There are many definitions of plastic size across different guidelines and articles. For consistency, the relative size of plastic ingested by fish in this study was sorted as microplastic (<5 mm), mesoplastic (5–25 mm), and macroplastic (25–1000 mm) [53,54,55]. For the shape of plastics, it was standardised into five categories: fibre, film, fragment, foam, and pellet (Table 1), which is in line with several studies [7,54,56,57,58]. The colours of the plastics were classified into red, orange, yellow, green, blue, purple, pink, brown, grey, black, white, transparent, and others. In studies that revealed plastics from the environment or other biota, only plastics ingested by fish were considered. If samples were collected from different environments, data from the same data were documented separately.

### 2.3. Statistical Analysis

Data of the number of plastic shape, colour, or polymer type (*k*) and the total number of plastics ingested (*n*) were extracted from the selected studies. Proportion of the plastic characterisation in a single study was calculated with the formula: *p = k/n*. Meta-analysis of proportions was employed to obtain a more precise estimation of the overall proportion for all plastic characterisations. Since proportions of <0.2 were common in the studies, the pooled prevalence of plastic characterisation was calculated by applying arcsine square root transformation on the proportion data. Publication bias was examined through funnel plots by trim-and-fill method and Egger’s regression test with a confidence interval (CI) of 95%. Between-study heterogeneity was evaluated with *I^2^* statistic and tested using the Paule-Mandel estimator method. Fixed effects model was used in the case of low heterogeneity whereas random effects model was used for high heterogeneity. Mixed effects meta-regression model was employed in which the random-effects model was used to combine study effects within each subgroup and the fixed-effect model was used to test if the effects across the subgroups differed significantly from each other. In this model, assumption of different between-study variance across subgroups was applied to identify if different moderators (i.e., sampling environment, plastic size, digestive organs examined, or sampling continent) affect the prevalence of the plastics. Subgroups forest plot was created based on different moderators. Meta-regression models were used to analyse characterisations that were the most abundant: shape (fibre, fragment, film, and pellet), colour (blue, black, transparent, and white), polymer types (polyethylene (PE), polyester (PES), polypropylene (PP), and polyamide (PA). The rare characterisations were not subtracted from the total plastic numbers even though they were not included in the meta-regression models. Hence, relative abundance of each characterisations were estimated based on total plastic numbers from all of its characterisations. All statistical analyses and plotting were performed in R software (R Core Team, version 4.1.2, Vienna, Austria). 

## 3. Results

### 3.1. Overview

The number of studies that reported the assessments of plastic size, shape, colour, and type were 127, 281, 195, and 153, respectively. Studies without QA/QC (*n* = 107) were excluded for the analysis of plastic size, while 94 studies with QA/QC and revealed the assessments of all three characterisations (shape, colour, and polymer type) in the same study were selected for meta-analysis. In total, data of five shapes, 13 colours, and 25 polymer types were recorded. It should be noted that the total count of plastics in polymer types was different from shape and colour, because not all of the plastics were tested with the polymer characterisation test.

### 3.2. Prevalence of Plastic Ingested

**Figure 2:**
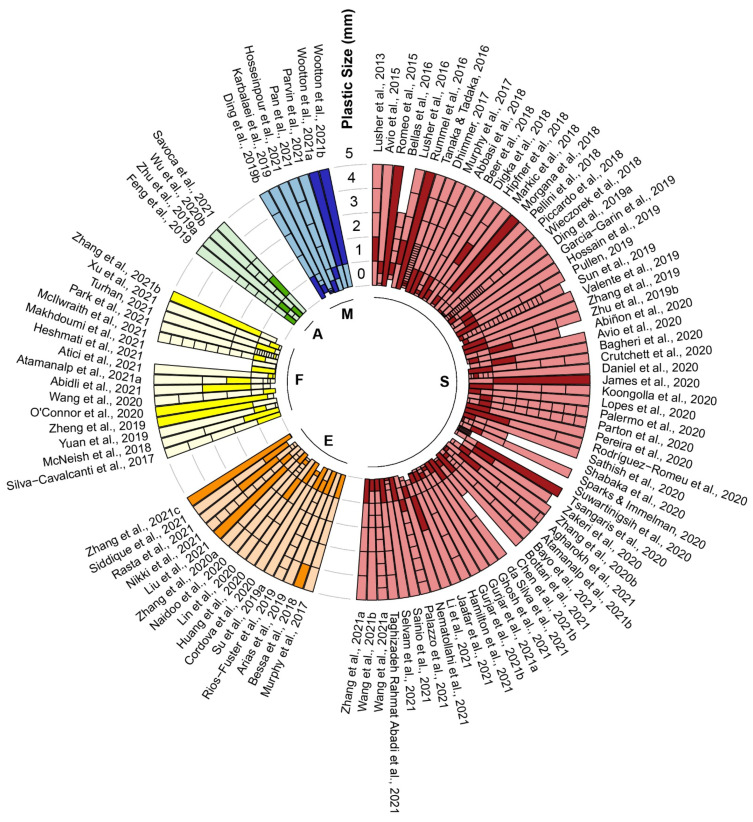
Overview of the assigned plastic size class and predominant size class of each study in different environments. Only size classes less than 5 mm are shown in this diagram. Each bar represents the plastic size class assigned in each study. Darker colour bars represent predominant size ingested. (S: Seawater; E: Estuarine; F: Freshwater; A: Aquaculture; M: Market). References: [27] Markic et al., 2018; [52] Ding et al., 2019a; [56] McNeish et al., 2018; [61] Abbasi et al., 2018; [62] Abidli et al., 2021; [63] Abiñon et al., 2020; [64] Agharokh et al., 2021; [65] Arias et al., 2019; [66] Atamanalp et al., 2021a; [67] Atamanalp et al., 2021b; [68] Atici et al., 2021; [69] Avio et al., 2015; [70] Avio et al., 2020; [71] Bagheri et al., 2020; [72] Bayo et al., 2021; [73] Beer et al., 2018; [74] Bellas et al., 2016; [75] Bessa et al., 2018; [76] Bottari et al., 2021; [77] Chen et al., 2021; [78] Cordova et al., 2020; [79] Crutchett et al., 2020; [80] da Silva et al., 2021; [81] Daniel et al., 2020; [82] Dhimmer, 2017; [83] Digka et al., 2018; [84] Ding et al., 2019b; [85] Feng et al., 2019; [86] Garcia-Garin et al., 2019; [87] Ghosh et al., 2021; [88] Gurjar et al., 2021a; [89] Gurjar et al., 2021b; [90] Hamilton et al., 2021; [91] Heshmati et al., 2021; [92] Hipfner et al., 2018; [93] Hossain et al., 2019; [94] Hosseinpour et al., 2021; [95] Huang et al., 2020; [96] Jaafar et al., 2021; [97] James et al., 2020; [98] Karbalaei et al., 2019; [99] Koongolla et al., 2020; [100] Li et al., 2021; [101] Lin et al., 2020; [102] Liu et al., 2021; [103] Lopes et al., 2020; [104] Lusher et al., 2013; [105] Lusher et al., 2016; [106] Makhdoumi et al., 2021; [107] McIlwraith et al., 2021; [108] Morgana et al., 2018; [109] Murphy et al., 2017; [109] Murphy et al., 2017; [110] Naidoo et al., 2020; [111] Nematollahi et al., 2021; [112] Nikki et al., 2021; [113] O’Connor et al., 2020; [114] Palazzo et al., 2021; [115] Palermo et al., 2020; [116] Pan et al., 2021; [117] Park et al., 2021; [118] Parton et al., 2020; [119] Parvin et al., 2021; [120] Pellini et al., 2018; [121] Pereira et al., 2020; [122] Piccardo et al., 2018; [123] Pullen, 2019; [124] Rasta et al., 2021; [125] Rios-Fuster et al., 2019; [126] Rodríguez-Romeu et al., 2020; [127] Romeo et al., 2015; [128] Rummel et al., 2016; [129] Sainio et al., 2021; [130] Sathish et al., 2020; [131] Savoca et al., 2021; [132] Selvam et al., 2021; [133] Shabaka et al., 2020; [134] Siddique et al., 2021; [135] Silva-Cavalcanti et al., 2017; [136] Sparks & Immelman, 2020; [137] Su et al., 2019; [138] Sun et al., 2019; [139] Suwartinigsih et al., 2020; [140] Taghizadeh Rahmat Abadi et al., 2021; [141] Tanaka & Tadaka, 2016; [142] Tsangaris et al., 2020; [143] Turhan, 2021; [144] Valente et al., 2019; [145] Wang et al., 2021a; [146] Wang et al., 2021b; [147] Wang et al., 2020; [148] Wieczorek et al., 2018; [149] Wootton et al., 2021a; [150] Wootton et al., 2021b; [151] Wu et al., 2020; [152] Xu et al., 2021; [153] Yuan et al., 2019; [154] Zakeri et al., 2020; [155] Zhang et al., 2020a; [156] Zhang et al., 2020b; [157] Zhang et al., 2019; [158] Zhang et al., 2021a; [159] Zhang et al., 2021b; [160] Zhang et al., 2021c; [161] Zheng et al., 2019; [162] Zhu et al., 2019a; [163] Zhu et al., 2019b.

**Figure 3:**
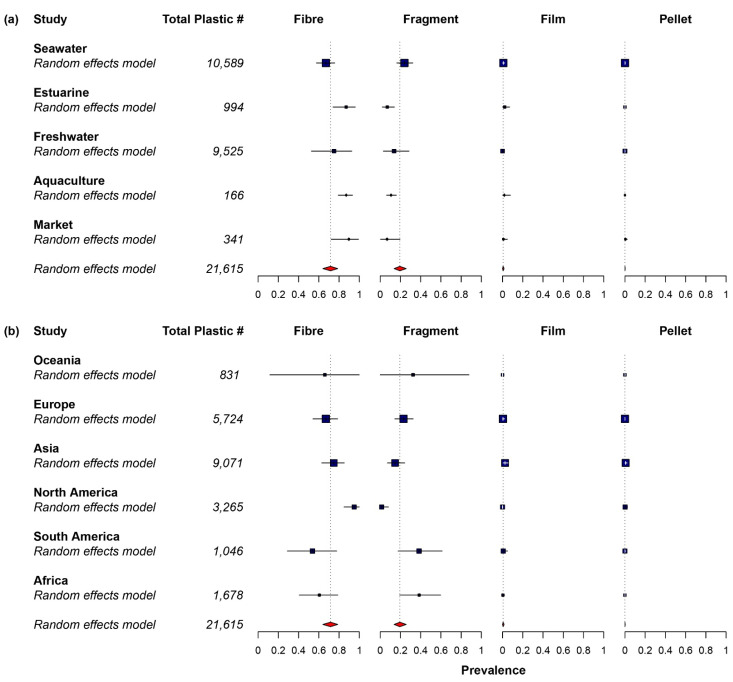
Prevalence forest plot for plastic shape. Blue squares represent subgroup means, while red diamonds and the dotted line represent the overall mean. (**a**) Subgroup of sampling environment. (**b**) Subgroup of sampling continent. For statistical details, see individual forest plots in supplementary information (Figures S4–S7).

**Figure 4:**
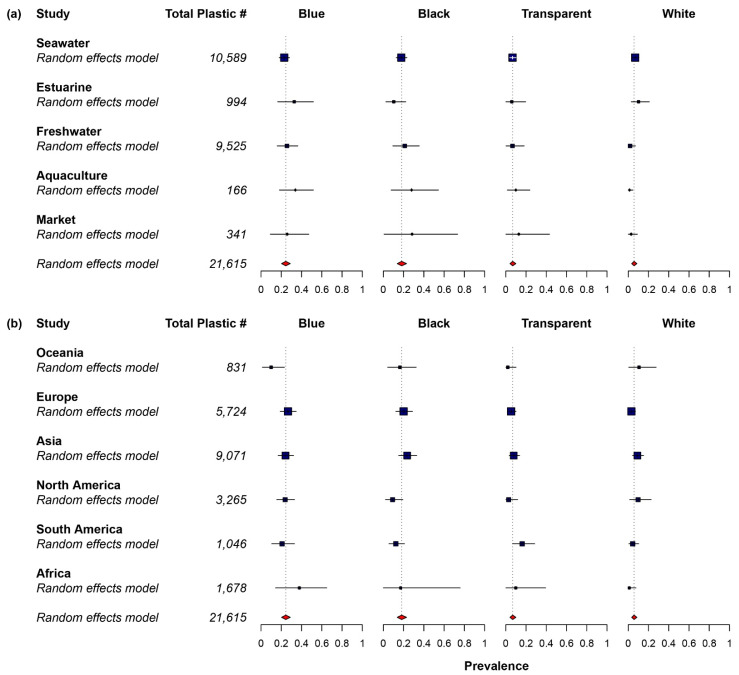
Prevalence forest plot for plastic colour. Blue squares represent subgroup means, while red diamonds and the dotted line represent the overall mean. (**a**) Subgroup of sampling environment. (**b**) Subgroup of sampling continent. For statistical details, see individual forest plots in supplementary information (Figures S8–S11).

**Figure 5:**
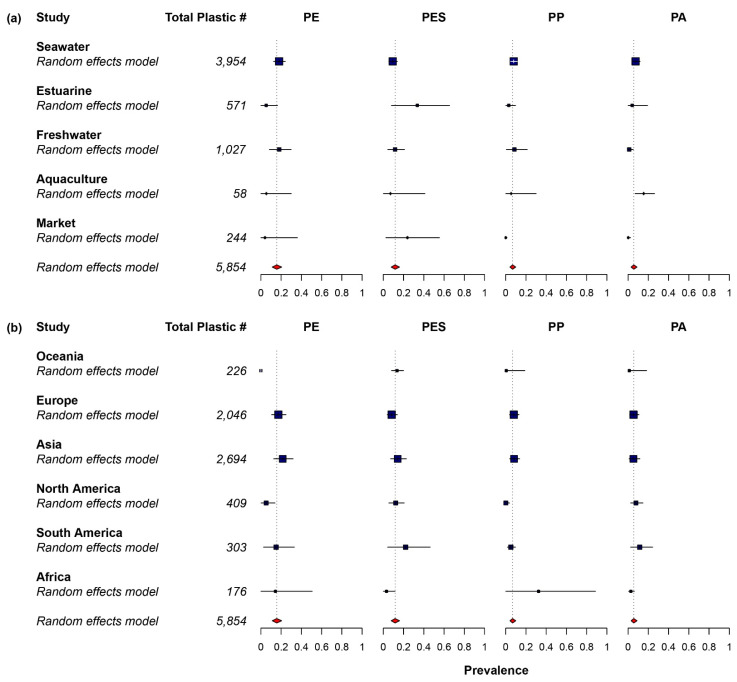
Prevalence forest plot for plastic polymer type. Blue squares represent subgroup means, while red diamonds and the dotted line represent the overall mean. (**a**) Subgroup of sampling environment. (**b**) Subgroup of sampling continent. PE: Polyethylene; PP: Polypropylene; PES: Polyester; PA: Polyamide. For statistical details, see individual forest plots in supplementary information (Figures S12–S15).

Only 34 out of the 107 studies (31.8%) included plastic sizes larger than 5 mm (mesoplastic and macroplastic) in their findings. Larger size particles were not included in many studies, especially recent studies, because they preferred to focus on microplastic ingestion. The most prevalent size of plastic ingested was microplastic for all the studies. Microplastics were often divided into two groups called small microplastic (<1 mm) and large microplastic (1–5 mm) [59,60]. Among the studies that reported the size class of plastic ingested, more than two-thirds of the studies (74.0%) recorded small microplastic as the predominant size class (Figure 2) [27,52,56,61,62,63,64,65,66,67,68,69,70,71,72,73,74,75,76,77,78,79,80,81,82,83,84,85,86,87,88,89,90,91,92,93,94,95,96,97,98,99,100,101,102,103,104,105,106,107,108,109,110,111,112,113,114,115,116,117,118,119,120,121,122,123,124,125,126,127,128,129,130,131,132,133,134,135,136,137,138,139,140,141,142,143,144,145,146,147,148,149,150,151,152,153,154,155,156,157,158,159,160,161,162,163]. Based on the pooled prevalence data, fibre plastic was the most abundant plastic ingested by the fish, with a relative abundance of 71.6% (CI 64.0–78.7%). The second most abundant plastic shape was fragment (19.4%; CI 13.8–25.7%), followed by film (0.5%; CI 0–1.5%) and pellet (0.0%; CI 0.0–0.2%) (Figure 3). Egger’s regression test indicated that there was no significant publication bias for plastic shapes (Figure S1, fragment: *Z* = 1.377, *p* = 0.169, pellet: *Z* = 1.491, *p* = 0.136) except fibre (*Z* = −2.256, *p* = 0.024) and film (*Z* = 2.457, *p* = 0.014). A high heterogeneity (*I^2^* = 93.6–98.8%) was observed between studies for plastic shapes. Furthermore, blue colour plastic was predominantly ingested by fish, with a relative abundance of 24.5% (CI 20.3–28.9%). The second most abundant plastic colour was black (18.1%; CI 13.7–22.9%), followed by transparent (6.8%; CI 4.1–9.9%), and white (5.8%; CI 3.4–8.5%) (Figure 4). Egger’s regression test revealed that there was no significant publication bias for plastic colours: blue (*Z* = 0.300, *p* = 0.764), black (*Z* = −0.050, *p* = 0.960), transparent (*Z* = 0.418, *p* = 0.676), and white (*Z* = −0.156, *p* = 0.876) (Figure S2). Similar to plastic shape, a high heterogeneity was found (*I^2^* = 98.0–98.6%) between studies on colour. The most abundant polymer type ingested by fish was PE, with a relative abundance of 15.7% (CI 11.3–20.6%), followed by PES (11.6%; CI 7.8–16.0%), PP (6.8%; CI 4.2–9.9%), and PA (5.6%; CI 2.9–8.8%) (Figure 5). Egger’s regression test indicated that there was no significant difference for polymer types: PE (*Z* = 0.738, *p* = 0.460), PES (*Z* = −0.560, *p* = 0.576), and PA (*Z* = −0.813, *p* = 0.416), except PP (*Z* = 2.128, *p* = 0.033) (Figure S3). The between-study heterogeneity for polymer types was slightly lower than plastic shape and colour (*I^2^* = 90.7–95.1%).

A similar proportion for the dominant class size was observed in different environments, except in estuary. Seawater environments had the largest percentage, with small microplastics as the predominant size class of plastic ingested (80.6%), followed by aquaculture (75.0%), market and freshwater (71.4%), and estuary (57.1%) (Figure 4). The subgroups of continents shared similar proportion, except in Oceania (50.0%). Asia had the largest proportion of small microplastics (77.6%), followed by North America and Africa (75.0%), and Europe (72.4%). A mixed-effects model was applied to identify potential sources of heterogeneity with four categorical moderators (sampling environment, plastic size, digestive organs examined, and sampling continent). A significant difference between groups was found for two out of the four moderators, specifically, environment and continent for plastic shape and polymer type. In the case of environment, a significant subgroup difference was observed in plastic shapes: fibre (*Q_m_* = 16.311, *p* = 0.003), fragment (*Q_m_* = 15.743, *p* = 0.003), and pellet (*Q_m_* = 16.453, *p* = 0.003), except in film (*Q_m_* = 0.824, *p* = 0.935). Fibre was relatively more abundant in the market (89.7%), estuary and aquaculture (87.0%) environments than in freshwater (75.0%) and seawater (67.0%) environments. In contrast, fragments were more abundant in seawater (23.9%) than in freshwater (13.7%), aquaculture (10.7%), estuary (7.0%), and market (6.8%). The continent groups appeared to be significantly different in plastic shapes: fibre (*Q_m_* = 18.734, *p* = 0.002), fragment (*Q_m_* = 24.886, *p* < 0.001), film (*Q_m_* = 28.279, *p* < 0.001), and pellet (*Q_m_* = 33.926, *p* < 0.001). The abundance of fibre was significantly higher in North America (95.0%, *p* = 0.001) than the rest of the continent: Asia (74.8%), Europe (66.9%), Oceania (66.0%), Africa (60.6%), and South America (53.7%). The prevalence of fragment was higher in Africa (38.5%), South America (38.4%), Oceania (32.5%), Europe (23.0%), and significantly lower in Asia (14.7%, *p* = 0.033), and North America (1.5%, *p* < 0.001).

For plastic colour, no significant subgroup difference was found in the moderator of environment, except white (*Q_m_* = 11.020, *p* = 0.026). The prevalence of blue plastic was highest in aquaculture (33.9%), followed by estuary (32.9%), market (25.8%), freshwater (25.6%), and seawater (22.9%) environments. In addition, the abundance of black plastic was higher in market (28.4%) and aquaculture (27.9%) than in freshwater (21.2%), seawater (17.7%), and estuary (10.3%) environments. Likewise, subgroup analysis with the moderator of continent revealed that there was no significant difference between plastic colours: blue (*Q_m_* = 5.156, *p* = 0.397), black (*Q_m_* = 5.936, *p* = 0.313), transparent (*Q_m_* = 5.259, *p* = 0.385), and white (*Q_m_* = 7.747, *p* = 0.188). In the moderator of environment, a significant difference was found in two polymer types, namely PP (*Q_m_* = 29.693, *p* < 0.001) and PA (*Q_m_* = 21.143, *p* < 0.001). PP had a higher abundance in freshwater (8.5%) and seawater (7.9%) than in aquaculture (5.4%), estuary (3.1%), and market (0%) environments. In contrast, PA was relatively more abundant in aquaculture (15.4%) than in seawater (7.4%), estuary (4.0%), freshwater (1.1%), and market (0.1%) environments. Subgroup analysis with the moderator of continent showed that a significant difference was found in PA (*Q_m_* = 50.287, *p* < 0.001) and PES (*Q_m_* = 12.174, *p* = 0.033). PE has the highest prevalence in Asia (21.6%), followed by Europe (17.2%), South America (15.1%), and Africa (14.3%), and significantly lower in North America (5.2%), and Oceania (0%). PES has a different distribution across continents, with a higher abundance in South America (22.0%), followed by Asia (14.2%), Oceania (13.6%), North America (12.2%), Europe (8.3%), and Africa (3.1%).

## 4. Discussion

Microplastics are widely defined as plastics with a size of <5 mm, whereas small microplastics and large microplastics are defined as plastics with a size of <1 mm and 1 to 5 mm, respectively. Small microplastics were the predominant plastic size ingested by fish in most of the reviewed studies. It was estimated that the most abundant plastic in the marine environment was microplastic (92.5%) [14]. The proportions of large and small microplastics in the marine environment were 62.3% and 37.7%, respectively. However, the concentration might be underestimated since the lower size limit of sampling and modelling used was 0.33 mm, whereby a 2.5-fold increase in microplastic contamination was observed when the lower size limit was 0.1 mm [164]. Hence, the actual concentration of small microplastics could be higher than the initial prediction. A similar concentration of microplastics can be expected in other environments since most of the microplastics in the marine environment originated from land sources such as sewage and runoff. A high concentration of small microplastics in the environment tend to be ingested by fish more easily through primary ingestion because they resemble their prey, especially zooplanktons, or secondary ingestion due to the attachment of plastics on their prey [15]. The predominance of small microplastics might be due to longer retention time in GIT, as they need longer time to be evacuated from the fish compared to larger size plastics [165]. However, several studies have excluded small microplastics during microscopic inspection and analysis, which might underestimate the actual number of plastics ingested [166,167,168,169]. It was reported that a lower detection limit would result in higher frequency of occurrence of plastic ingestion [46]. Studies with fish samples of smaller body size may influence the outcome, since they are unable to ingest larger size plastics. Therefore, there is a need to reduce the threshold size of plastic detection in order to identify all plastics, since small microplastics dominate the plastic ingested.

This meta-analysis showed that the largest percentage of plastics ingested by fish was in the form of fibre and fragment. Several studies have documented fibre plastics to be the most prevalent type of plastic in seawater, freshwater, and aquaculture environments [170,171,172,173,174]. Fibre plastics in the environment originate mainly from the effluent of wastewater treatment plants. An experiment illustrated that a single garment is able to produce >1900 fibres per wash and all garments can release >100 fibres per litre of effluent [12]. Similarly, it was estimated that over 700,000 fibres could be discharged from an average wash load of 6 kg fabrics [175]. Another source of fibre plastic in the environment could be from the fishery activities. The abrasion of abandoned, lost, or discarded fishing gears has contributed about 18% of the marine plastic debris in the marine environment [4]. Some fish species do not actively take up fibre plastic; instead, the fibre plastics are passively sucked in while breathing [176]. Therefore, most of the fish species may unintentionally ingest plastics that are ubiquitous in the environment. After exposure to microplastic in a laboratory study, fibre plastic accumulated the most in the gut of zebrafish, followed by fragment and pellet plastics [177]. Another study demonstrated that fibre and pellet plastics shared a similar retention time in the GIT when goldfish were fed with plastic of different shapes [178]. Shape-dependent accumulation of plastic could be another factor contributing to the prevalence of fibre plastic in fish, but more research is required. The accumulation period of plastic in GIT of fish may affect the outcome of the studies, as the plastics that have been extracted from the fish do not exactly represent the amount of plastic ingested throughout its lifetime. Instead, those samples that were found to have a relatively smaller quantity of non-fibre plastic might have egested those plastics out of their bodies when they were sampled. Hence, a larger sample size of the same species from the same sampling area should be examined to tackle this limitation.

Among the studies reviewed, blue is the most common plastic colour ingested by fish, followed by black, white, and transparent. Based on the global analysis of floating plastics in sea water, white and transparent/translucent (47%) are the most abundant plastic colours, followed by yellow and brown (26%), and blue (9%) [179]. This does not imply that the plastics in the ocean are mostly white and transparent/translucent, as the authors have excluded fibre plastic from the analysis due to the possibility of airborne contamination and fragments made up 83.6% of all the plastics collected. For studies that included fibre plastic, the predominant colours of the fibre were blue, black, transparent, and white [170]; black, grey, blue, and red [180]; transparent, blue, black, and red [181]; and transparent, white, blue, and red [182], respectively. The inconsistent results among the studies could be attributed to the differences in methodology and sampling region. Similar dominant colours such as blue, black, white, and transparent were observed in different studies. Hence, fish might accidentally consume the plastics by feeding or breathing, since the results were similar to the colour of plastics present in the environment. A study conducted in the China Sea revealed that the proportion of the plastic colour ingested by fish was similar to the proportion in water and sediment of the same sampling site [156]. Another possible explanation for the results could be related to selective feeding for the species sampled. Large pieces of plastic debris with blue and yellow colours were reported to be preferred by the fish [183]. Blue plastics were found to be predominantly ingested by Amberstripe scad, Atlantic chub mackerel, and fish larvae due to the resemblance to one of their preys: blue pigmented copepod species that were abundant in the sampling areas [184,185,186]. The blue pigmentation featured on zooplankton in the ocean [187] might account for them being confused with blue plastic particles. We hypothesise that only specific fish species ingest blue plastic deliberately due to the resemblance to its prey and most species consume blue plastic incidentally as a result of its abundance during feeding and breathing.

Our results confirmed that PE, PES, PP, and PA were the most prevalent polymer types ingested by fish globally. The results were not surprising, as these polymer types were widely found in marine and freshwater environments [173,188,189]. The abundance of these polymer types in the environments could be due to improper disposal of plastic waste, as they accounted for 80% of the global plastic waste generated in 2015 [190]. PE and PP might be derived from the abrasion of fishing tools, since they are widely used in fishery activities around the world, as well as the packaging used for foods and manufactured products. PE and PP are less dense polymers that will usually float on the surface of the water and are likely to be ingested by pelagic species, while demersal species tend to ingest dense plastics such as PES and PA because they usually suspend in the water column or deposition in the seabed. PA and PES are widely used in fishery activities and the clothing industry. The abundance of PA and PES in the environment is mostly originated from the effluent of washing clothes and the usage of fishery tools. For some studies, only part of the plastics extracted from the samples was tested with the polymer characterisation test, which could lead to a potential bias of these results.

## 5. Gaps and Recommendations

Fish are an essential component of a healthy human diet. Fish consumption increased significantly from 9.0 kg per capita in 1961 to 20.5 kg per capita in 2018 worldwide, which increased at an average annual rate of 1.5% [191]. As of 2017, fish consumption contributed 17% of animal protein intake, and 7% of all protein intake globally [191]. Although the viscera of fish are removed prior to consumption, humans still have a strong likelihood to be exposed to microplastics and even nanoplastics (<1 µm) due to the translocation of plastics to muscle tissues [192]. Meanwhile, many commercial fish species have been found to have microplastics embedded in their muscles, which are likely to be consumed by humans [61,193,194]. It was reported that seafood was one of the top three contributors of microplastics consumption by humans among the commonly consumed items [195]. Fish and bivalves were the seafood included in the study and they estimated that the total microplastics consumption of a person ranged from 39,000 to 52,000 particles per year. Lately, microplastics were detected within a small sample size of human stools, suggesting that humans had ingested these particles [196,197]. Although there was no direct evidence showing the sources of microplastics ingested by humans, it is still highly possible that part of the microplastics ingested originated from seafood, since the majority of the participants in the study consumed seafood within the study period [196,197]. Nevertheless, some fish species such as Japanese anchovy are commonly consumed by humans without the elimination of GIT, and it further increases the risk of translocation of plastic from fish to humans [141]. Furthermore, 262 out of 391 species that ingested plastic are commercial species that are frequently consumed by humans [44]. This should raise awareness of the dangers of consuming microplastics, since it poses a significant threat to human health [198]. However, research concerning plastic ingestion of fish in aquaculture environments has been overlooked and there are only a few studies on the incidence of plastic ingestion within this environment [151,162,199,200,201]. As of 2018, the contribution of world aquaculture to global fish production reached 82.1 million tonnes annually, which contributed 46.0% of the total fish production and increased from 25.7% in 2000 [191]. Fish cultured in aquaculture are exposed to plastic debris due to aged and shattered fishery equipment [202] and to contaminated feeds [203]. In fact, aquaculture sites are prone to accumulate plastic debris that may be ingested by fish incidentally [151,162]. There are studies showing that aquaculture fish have a lower incidence plastic ingestion than wild fish [200,201]. Hence, awareness towards them should be raised to further investigate the plastic contamination level within aquaculture fish, since they constitute almost half of the fish for human consumption globally.

Furthermore, gill and muscle tissue of the same sample should be examined together for the presence of plastic, since plastic contamination in gill was often reported [61,204] and even poses health risk towards the fish [205]. Deficiency of the record of plastic ingestion by fish is evident, as only 555 out of 22,581 known species have been investigated [46,206], comprising 2.5% compared to other taxa such as sea birds (44.0%), marine mammals (56.1%), and turtles (100.0%) [207]. Although there has been a significant improvement in ingestion records compared to previous records (fish, 0.3%; sea birds, 39.1%; marine mammals, 26.1%; and turtles, 85.7%) [49], more research on plastic ingestion in other fish species is necessary to further reveal the potential hazards in the environment.

In future research, the lowest threshold of plastic size should be mentioned in the study and threshold filter pore size must be at least 1 µm to fulfil the criteria of microplastics [208] and to capture all plastics ingested, since the predominant size of the plastic is <1 mm. It is difficult to compare the dominant size class ingested by fish across different studies because most of the studies have assigned a distinct size class (Figure 2), and the inconsistent classifications have made the comparison of plastic ingested by size more difficult. Instead, the plastic size classes should be standardised for ease of comparison of the dominant size class of plastic ingested between studies. Likewise, the shape of the plastics should be standardised, as suggested by GESAMP [54], into fibre, fragment, film, pellet, and foam. Since fibre is the dominant plastic shape ingested by fish, it should not be excluded from the analysis. Possible contamination should not be used as an exclusion criterion for plastic analysis [209]. Instead, extra care should be taken to eliminate possible contamination [210]. For studies that intend to investigate only the occurrence of microplastic in fish, any plastic that is 5 mm and above should not be excluded [211]; instead, it should be archived to record their characterisations such as size, shape, and colour, since it is still an anthropogenic particle and may pose a significant risk towards the fish. Polymer identification tests should be carried out randomly among the plastics extracted from the samples [212]. For future studies, it is essential that the size, colour, and shape of plastic ingestion be recorded and analysed to further validate if the fish species has a certain preference regarding plastic ingestion.

## 6. Conclusions

Our meta-analysis has revealed that the most abundant plastics ingested by fish globally was <1 mm in size, fibre shape, blue colour, and PE polymer. The results obtained were similar to the prevalence of plastics in environments where most of the fish species could ingest them passively. Hence, more research needs to be carried out in order to further validate if fish have a certain preference for ingesting plastic particles. Since fish are a one of the major protein sources, the incidence of plastic ingestion by fish, especially in aquaculture sites, should be a major cause for alarm, as it poses potential threats to human health, yet there is still a lack of information on plastic ingestion in many commercial fish species. Furthermore, it is essential that a standardised classification of plastic size, shape, and colour be established for use in future studies. A better understanding of the causes of plastic ingestion by fish can be achieved by adapting a uniform classification of plastic characterisations.

## Figures and Tables

**Table 1:** Standardised shape description of plastic.

Standardised Shape	Description	Alternative Shape
Fibre	Thin or fibrous plastic that has a length longer than its width	Line, Monofilament, Thread, Polyfilament, Twine, Fibrous, Microfibre
Film	Flat and thin plane of smooth or angular edges plastic	Sheet, Plastic Packaging, Wrapper, Plastic Bag, Packet Wrap, Food Package, Strip
Fragment	Irregular, hard, and jagged plastic particle	Flake, Particle, Piece, Tag, Chip
Foam	Lightweight, sponge-like plastic	Polystyrene, Polystyrene Spherule, Styrofoam, Styrofoam Fragment, Sponge, Expanded Polystyrene Foam (EPS)
Pellet	Hard, rounded plastic particle	Bead, Granule, Microbead, Particle, Spherule

Note: Particle shape of each study was assigned to the closest standardised shape based on the appearance shown in the publications.

## Data Availability

Not applicable.

## Supplementary Materials

The following supporting information can be downloaded at: https://www.mdpi.com/article/10.3390/toxics10040186/s1, Figure S1: Funnel plot for the prevalence of plastic’s shapes ingested by fish from all environments. Studies are represented by full circles and imputed studies are represented by empty circles, Figure S2: Funnel plot for the prevalence of plastic’s colours ingested by fish from all environments. Studies are represented by full circles and imputed studies are represented by empty circles, Figure S3: Funnel plot for the prevalence of plastic’s polymer type ingested by fish from all environments. Studies are represented by full circles and imputed studies are represented by empty circles. PE: Polyethylene; PP: Polypropylene; PES: Polyester; PA: Polyamide; PS: Polystyrene, Figure S4: Forest plot for fibre subgroup analysis. Red diamonds represent subgroup means. Total: total plastics found in each study. Fibre: number of fibres found in each study, Figure S5: Forest plot for fragment subgroup analysis. Red diamonds represent subgroup means. Total: total plastics found in each study. Fragment: number of fragments found in each study, Figure S6: Forest plot for film subgroup analysis. Red diamonds represent subgroup means. Total: total plastics found in each study. Film: number of films found in each study, Figure S7: Forest plot for pellet subgroup analysis. Red diamonds represent subgroup means. Total: total plastics found in each study. Pellet: number of pellets found in each study, Figure S8: Forest plot for blue subgroup analysis. Red diamonds represent subgroup means. Total: total plastics found in each study. Blue: number of blues found in each study, Figure S9: Forest plot for black subgroup analysis. Red diamonds represent subgroup means. Total: total plastics found in each study. Black: number of blacks found in each study, Figure S10: Forest plot for transparent subgroup analysis. Red diamonds represent subgroup means. Total: total plastics found in each study. Transparent: number of transparent found in each study, Figure S11: Forest plot for white subgroup analysis. Red diamonds represent subgroup means. Total: total plastics found in each study. White: number of whites found in each study, Figure S12: Forest plot for PE subgroup analysis. Red diamonds represent subgroup means. Total: total plastics found in each study. PE: number of PE found in each study. PE: Polyethylene, Figure S13: Forest plot for PES subgroup analysis. Red diamonds represent subgroup means. Total: total plastics found in each study. PES: number of PES found in each study. PES: Polyester, Figure S14: Forest plot for PP subgroup analysis. Red diamonds represent subgroup means. Total: total plastics found in each study. PP: number of PP found in each study. PP: Polypropylene, Figure S15: Forest plot for PA subgroup analysis. Red diamonds represent subgroup means. Total: total plastics found in each study. PA: number of PA found in each study. PA: Polyamide.
