# Supplementary material for: A Meta-Analysis of the Characterisations of Plastic Ingested by Fish Globally
Source: Toxics. 2022 Apr 11;10(4):186. doi: 10.3390/toxics10040186 (PMC9027263; doi:10.3390/toxics10040186)
Supplement: Supplementary file 1 [file toxics-10-00186-s001.zip › toxics-1668365-supplementary.pdf]

toxics

Supplementary Mterials: A Meta-Analysis of the Characterisations of Plastic Ingested by Fish Globally

Kok Ping Lim, Phaik Eem Lim, Sumiani Yusoff, Chengjun Sun, Jinfeng Ding and Kar Hoe Loh

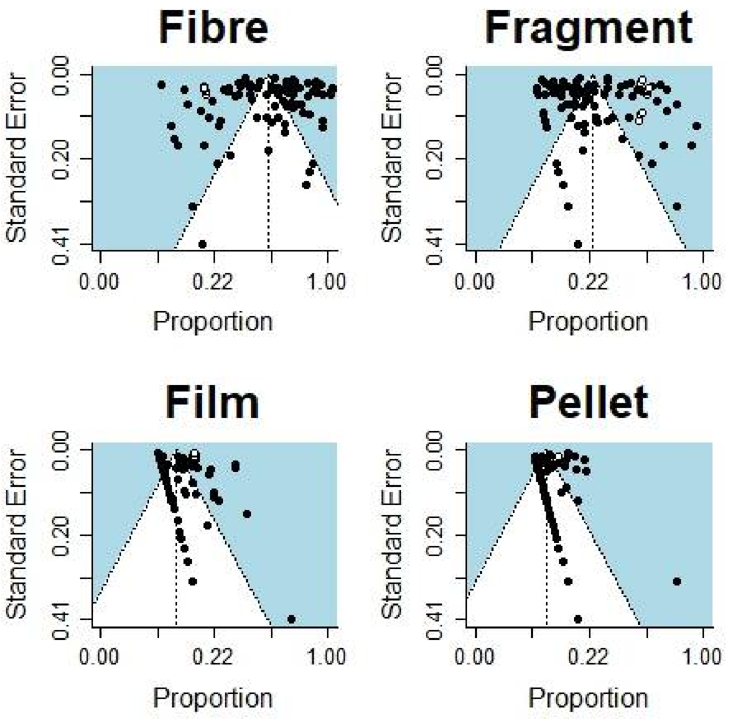

Figure S1. Funnel plot for the prevalence of plastic's shapes ingested by fish from all environments. Studies are represented by full circles and imputed studies are represented by empty circles.

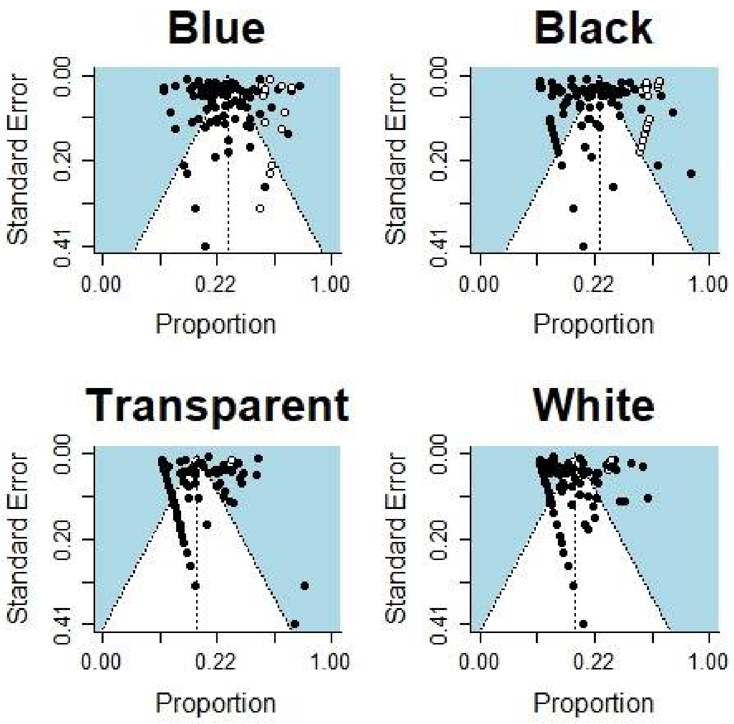

Figure S2. Funnel plot for the prevalence of plastic's colours ingested by fish from all environments. Studies are represented by full circles and imputed studies are represented by empty circles.

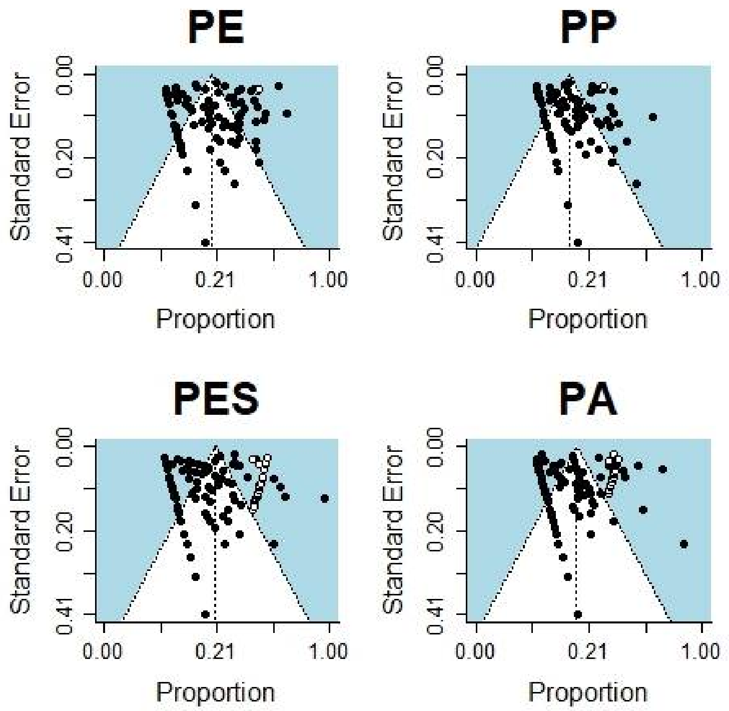

Figure S3. Funnel plot for the prevalence of plastic's polymer type ingested by fish from all environments. Studies are represented by full circles and imputed studies are represented by empty circles. PE: Polyethylene; PP: Polypropylene; PES: Polyester; PA: Polyamide; PS: Polystyrene.

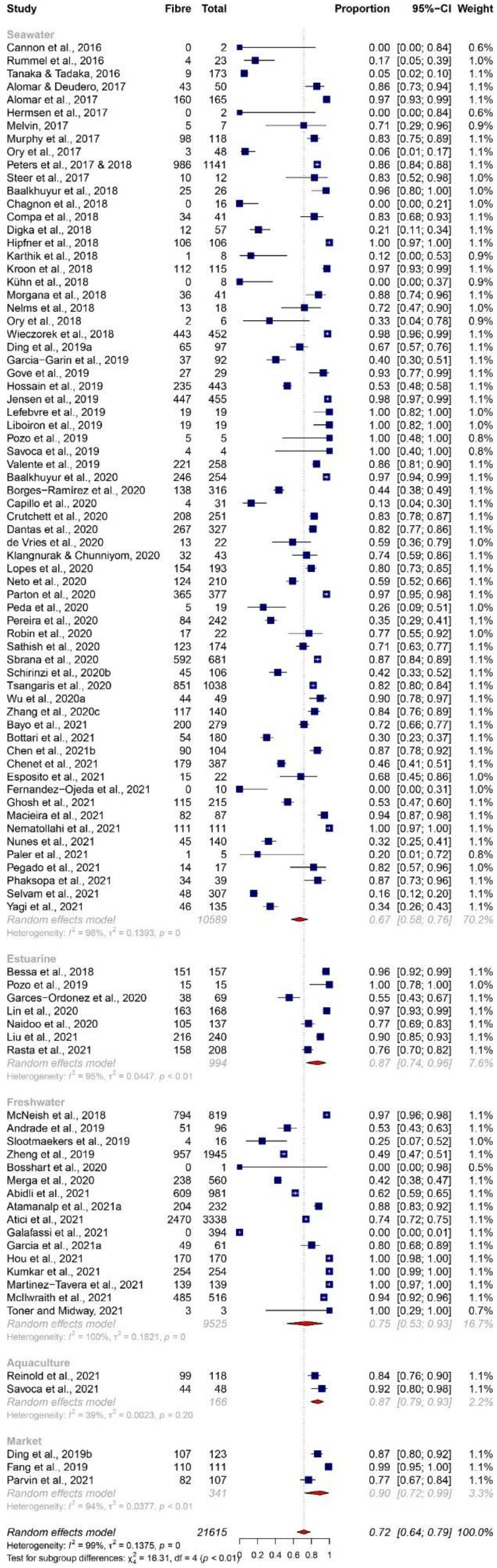

Figure S4. Forest plot for fibre subgroup analysis. Red diamonds represent subgroup means. Total: total plastics found in each study. Fibre: number of fibre found in each study.

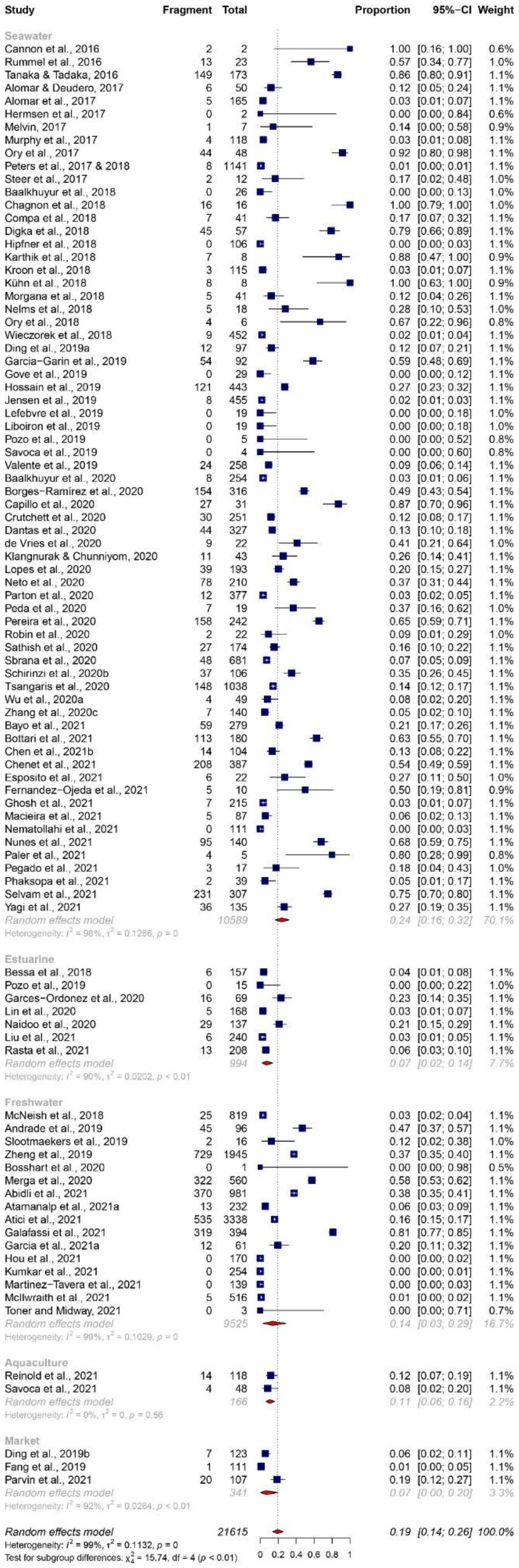

Figure S5. Forest plot for fragment subgroup analysis. Red diamonds represent subgroup means. Total: total plastics found in each study. Fragment: number of fragment found in each study.

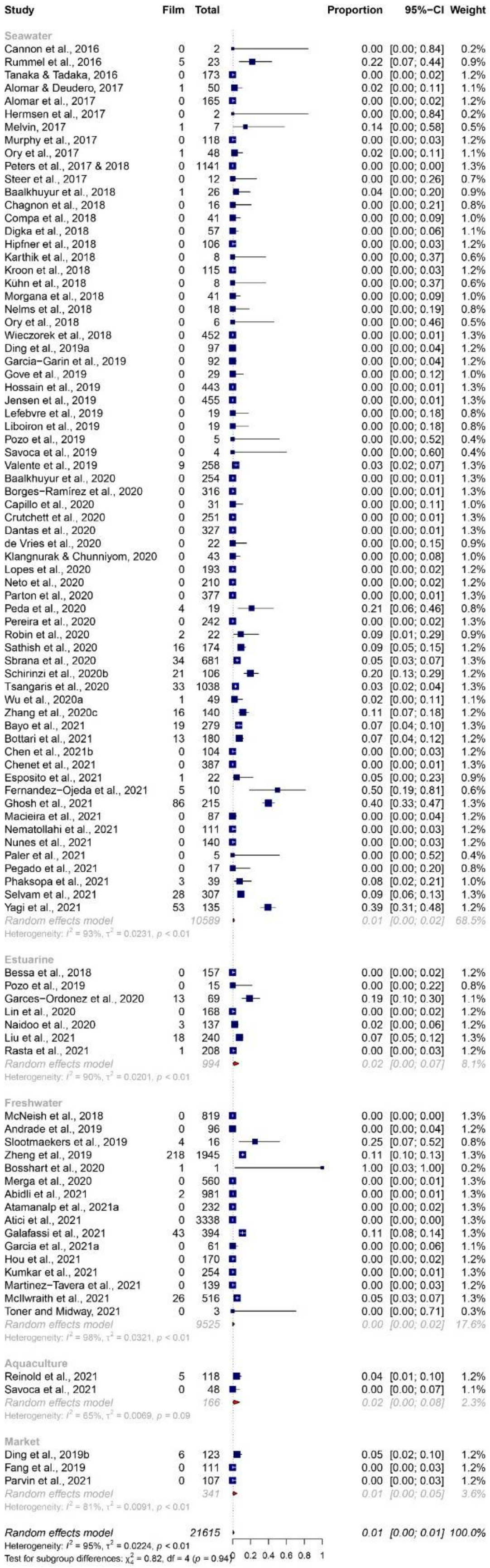

Figure S6. Forest plot for film subgroup analysis. Red diamonds represent subgroup means. Total: total plastics found in each study. Film: number of film found in each study.

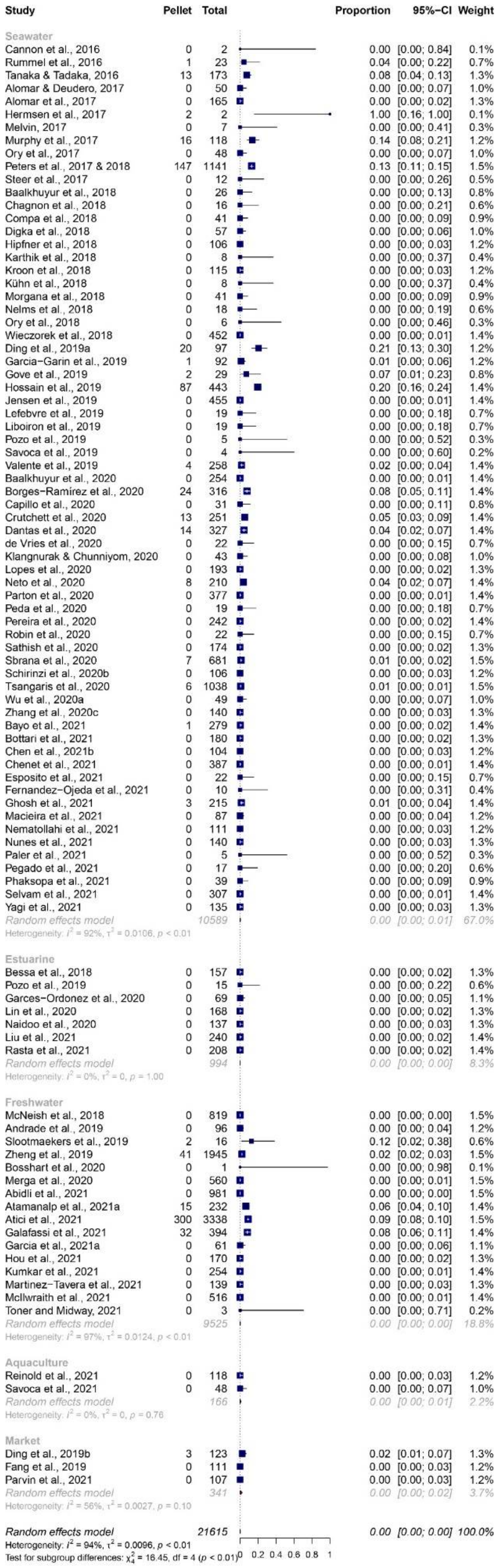

Figure S7. Forest plot for pellet subgroup analysis. Red diamonds represent subgroup means. Total: total plastics found in each study. Pellet: number of pellet found in each study.

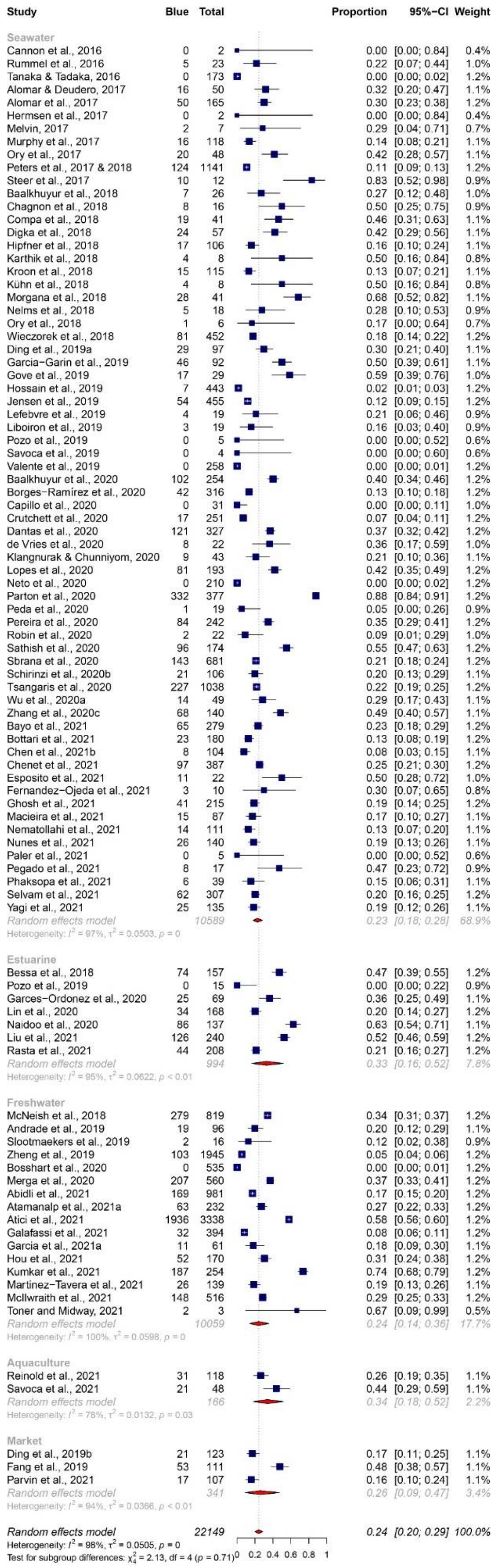

Figure S8. Forest plot for blue subgroup analysis. Red diamonds represent subgroup means. Total: total plastics found in each study. Blue: number of blue found in each study.

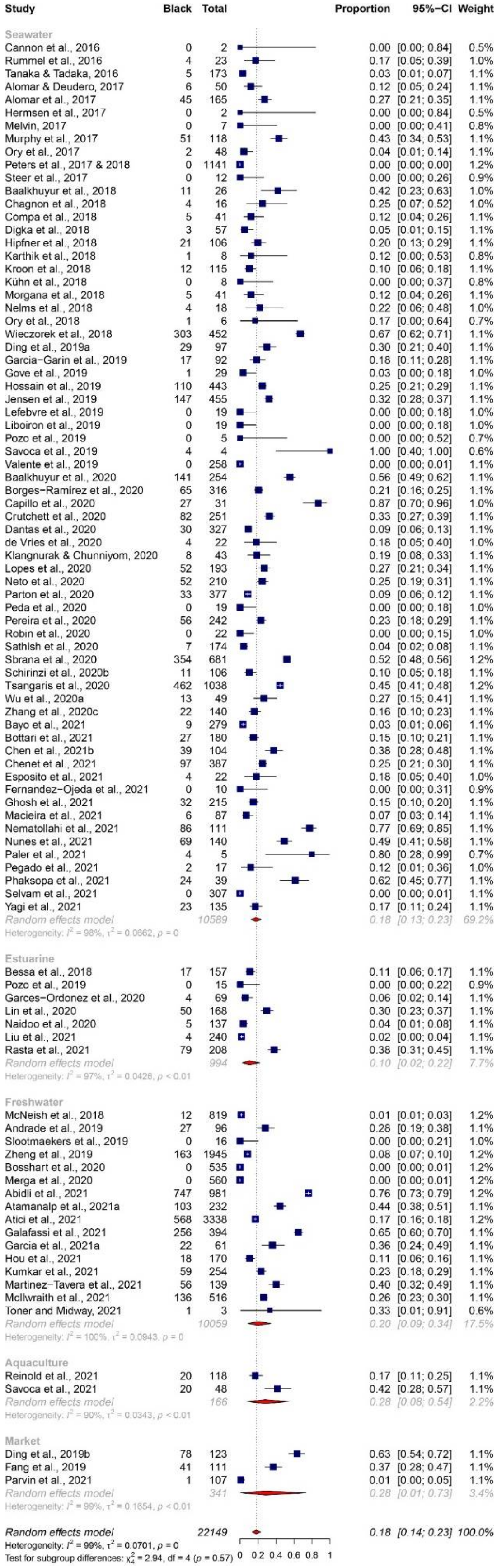

Figure S9. Forest plot for black subgroup analysis. Red diamonds represent subgroup means. Total: total plastics found in each study. Black: number of black found in each study.

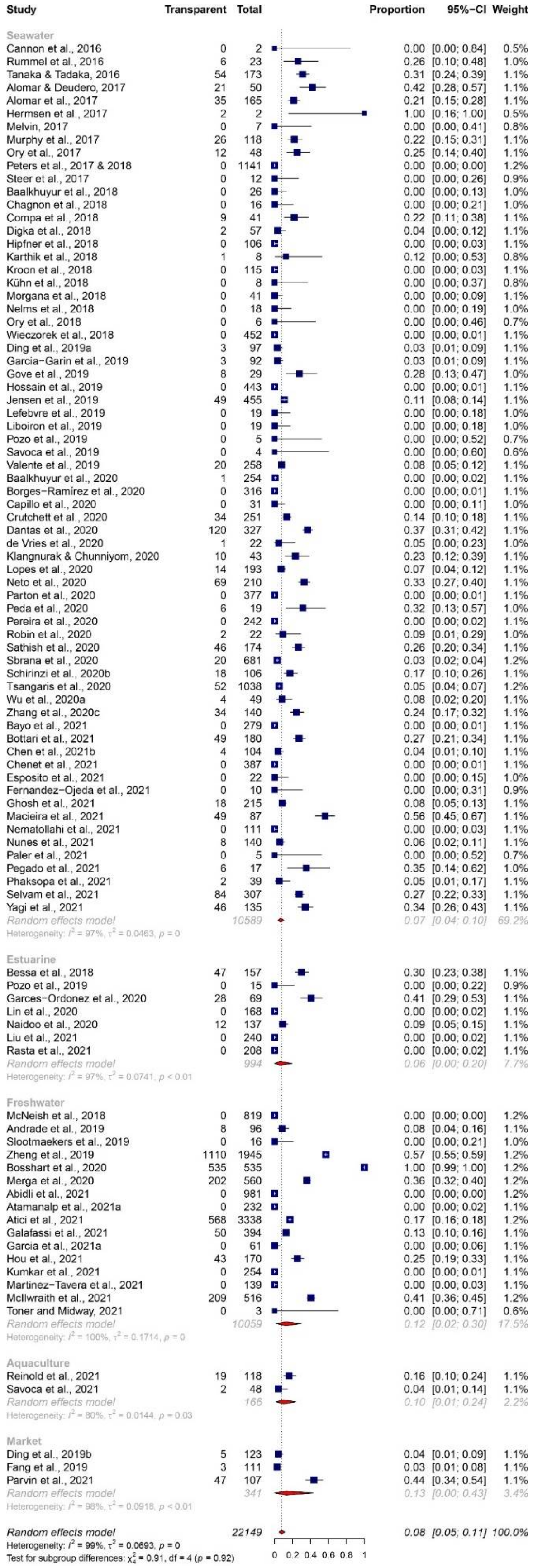

Figure S10. Forest plot for transparent subgroup analysis. Red diamonds represent subgroup means. Total: total plastics found in each study. Transparent: number of transparent found in each study.

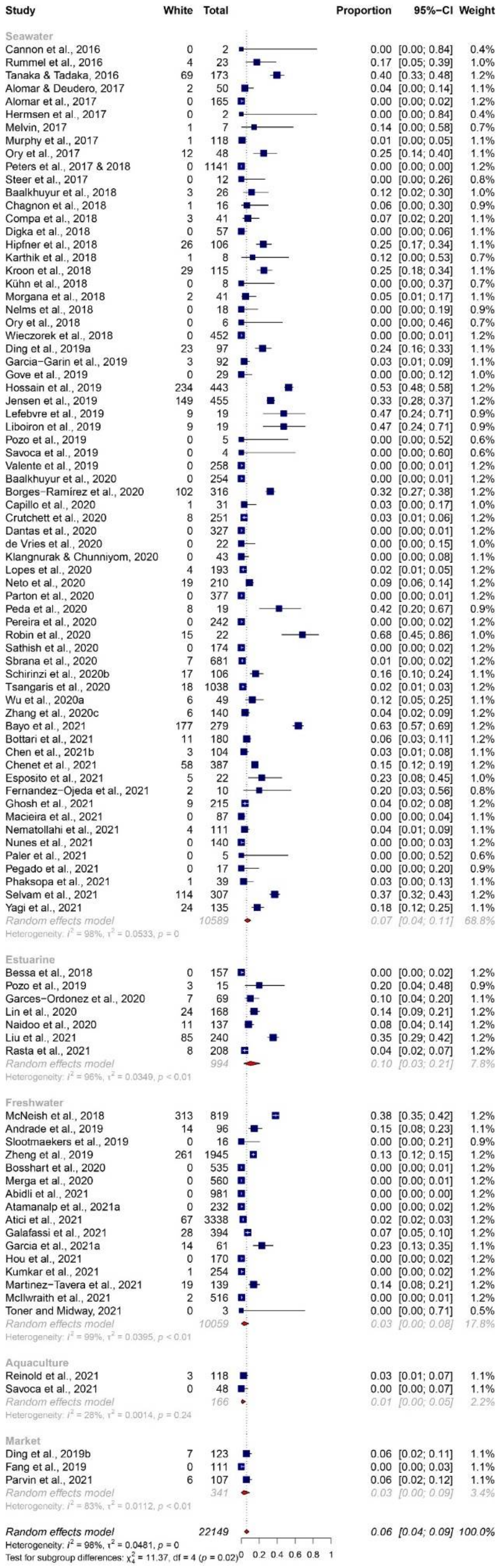

Figure S11. Forest plot for white subgroup analysis. Red diamonds represent subgroup means. Total: total plastics found in each study. White: number of white found in each study.

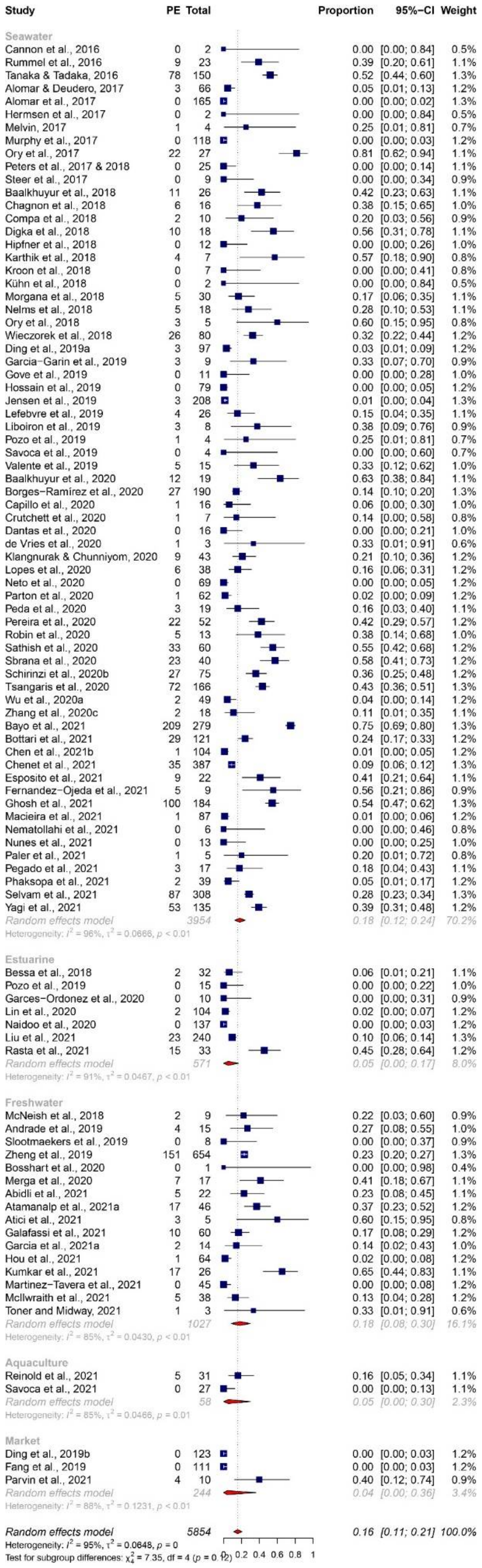

Figure S12. Forest plot for PE subgroup analysis. Red diamonds represent subgroup means. Total: total plastics found in each study. PE: number of PE found in each study. PE: Polyethylene.

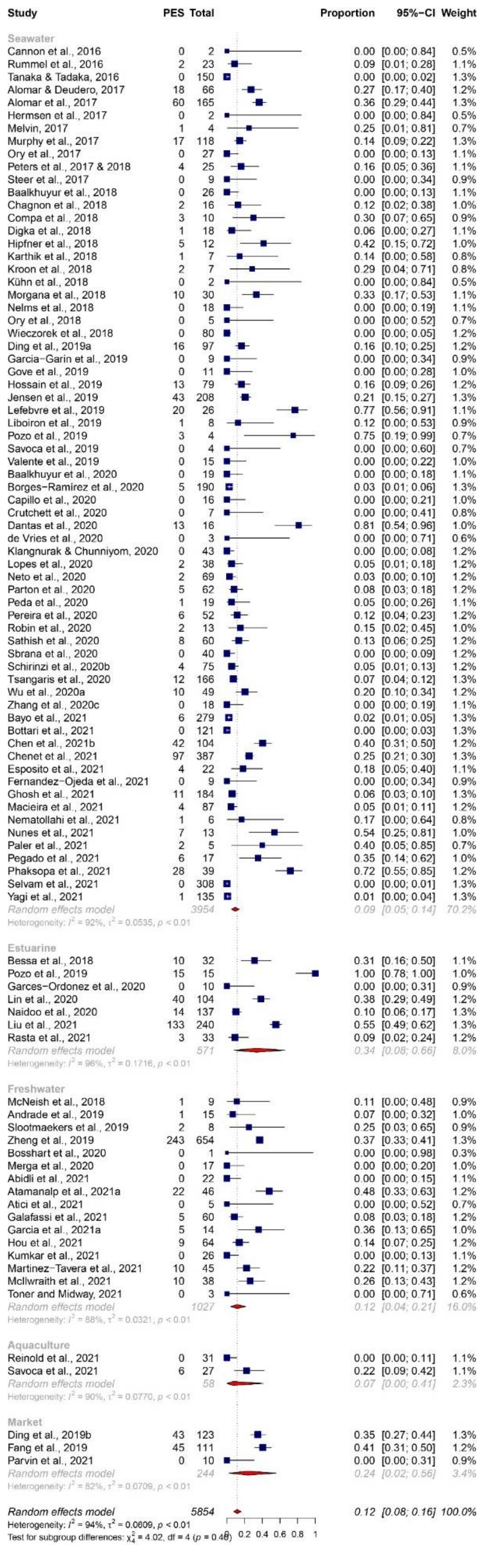

Figure S13. Forest plot for PES subgroup analysis. Red diamonds represent subgroup means. Total: total plastics found in each study. PES: number of PES found in each study. PES: Polyester.

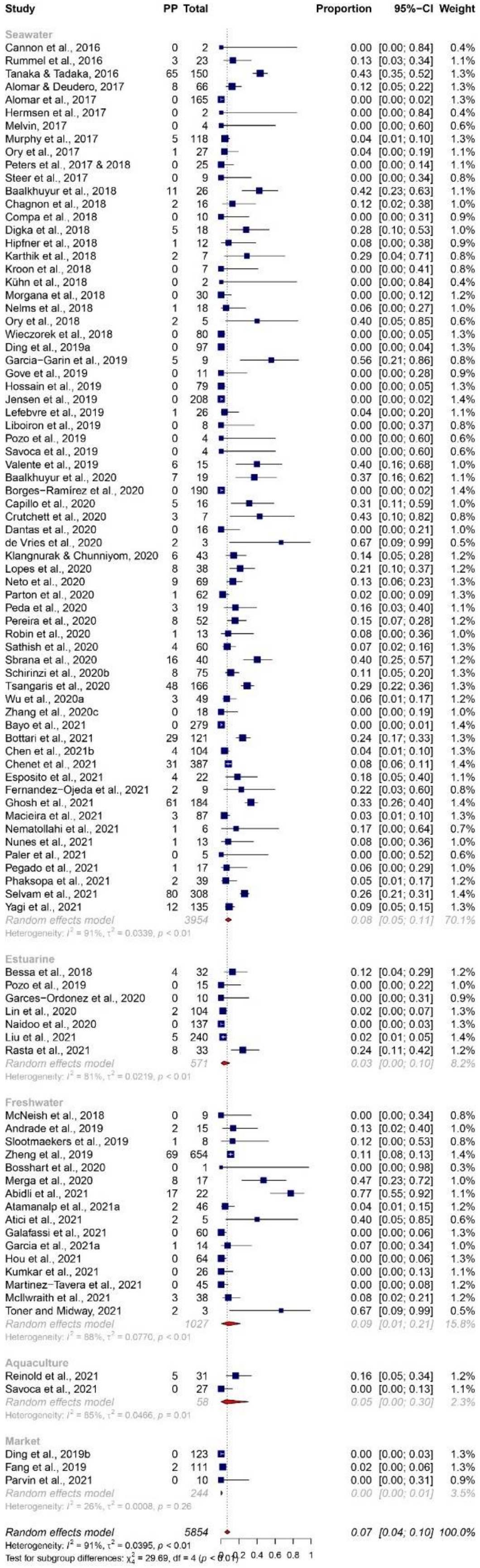

Figure S14. Forest plot for PP subgroup analysis. Red diamonds represent subgroup means. Total: total plastics found in each study. PP: number of PP found in each study. PP: Polypropylene.

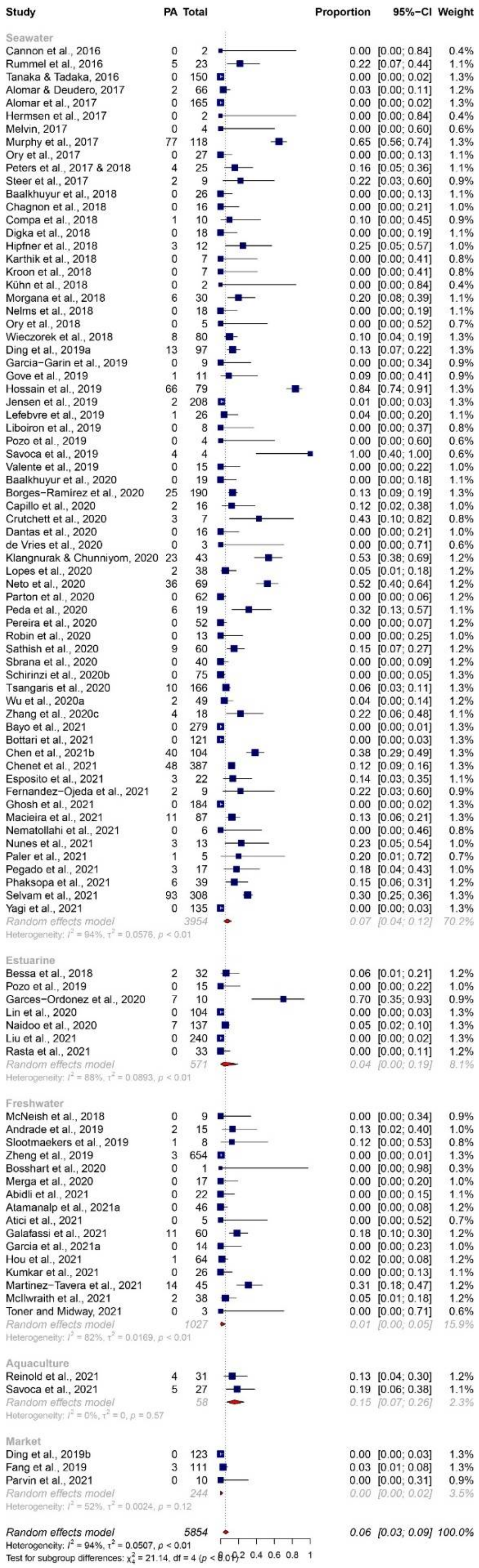

Figure S15. Forest plot for PA subgroup analysis. Red diamonds represent subgroup means. Total: total plastics found in each study. PA: number of PA found in each study. PA: Polyamide.
